# Human Papillomavirus and Risk of Head and Neck Squamous Cell Carcinoma in Iran

**DOI:** 10.1128/spectrum.00117-22

**Published:** 2022-06-16

**Authors:** Abbas Karimi, Elham Mohebbi, Sandrine Mckay-Chopin, Hamideh Rashidian, Maryam Hadji, Vahideh Peyghambari, Maryam Marzban, Ahmad Naghibzadeh-Tahami, Mahin Gholipour, Farin Kamangar, Massimo Tommasino, Tarik Gheit, Kazem Zendehdel

**Affiliations:** a Department of Molecular Medicine, Faculty of Advanced Medical Sciences, Tabriz University of Medical Sciences, Tabriz, Iran; b Early Detection, Prevention and Infections Branch, International Agency for Research on Cancer (IARC), Lyon, France; c Cancer Research Center, Cancer Institute, Tehran University of Medical Sciences, Tehran, Iran; d Pathology and Stem Cell Research Center, Kerman University of Medical Sciences, Kerman, Iran; e Health Sciences Unit, Faculty of Social Sciences, Tampere University, Tampere, Finland; f Department of Public Health, School of Public Health, Bushehr University of Medical Science, Bushehr, Iran; g Clinical Research Development Center, The Persian Gulf Martyrs, Bushehr University of Medical Science, Bushehr, Iran; h Social Determinants of Health Research Center, Institute for Future Studies in Health, Kerman University of Medical Sciences, Kerman, Iran; i Department of Biostatistics and Epidemiology, Kerman University of Medical Sciences, Kerman, Iran; j Metabolic Disorders Research Center, Golestan University of Medical Sciences, Gorgan, Iran; k Department of Biology, School of Computer, Mathematical, and Natural Sciences, Morgan State University, Baltimore, Maryland, USA; l Cancer Biology Research Center, Cancer Institute, Tehran University of Medical Sciences, Tehran, Iran; University of Manitoba

**Keywords:** alpha papillomavirus, beta papillomavirus, gamma papillomavirus, head and neck squamous cell carcinoma, Iran

## Abstract

Human papillomavirus (HPV) causes a subset of head and neck squamous cell carcinoma (HNSCC). Knowledge of determinants of α-, β-, and γ-HPVs types in the oral cavity is required for a better understanding of HNSCC development. Oral rinse samples of 498 HNSCC cases and 242 controls from the IROPICAN study—a large multicenter case-control study in Iran—were screened for 21 α-HPV, 46 β-HPVs, and 52 γ-HPVs using bead-based HPV genotyping assays. α-HPVs were detected only in 1.2% of the patients and 2.9% of the controls from which HPV16 was the most prevalent type among participants. β-HPVs were detected in 43.8% of the patients and 38.6% of the controls where the lip and oral cavity (45.5%) had the highest positivity. Values for γ-HPV prevalence in patients and controls were 26.1% and 24.7%, respectively. The highest percentage of γ-HPV positivity was found in the larynx (30.4%). Concerning the β genus, HPV23 and HPV38 were the most prevalent types among the patients and controls, respectively. For the γ genus, SD2 in cases and HPV134 in controls were the most prevalent types. Overall, detection of α-HPVs (aOR, 0.40; 95% CI = 0.1 to 1.2; *P* = 0.11), β-HPVs (aOR, 1.9; 95% CI = 0.9 to 1.6; *P* = 0.29), and γ-HPVs infections (aOR, 1.04; 95% CI = 0.7 to 1.5; *P* = 0.83) was not associated with the HNSCC development. Our data did not suggest an HPV-related etiology for HNSCC pathogenesis. Nonetheless, this study provides novel insights into the diversity of β-, and γ-HPVs in different HNSCC anatomical subsites.

**IMPORTANCE** Infection with human papillomavirus (HPV) is responsible for a subset of neck squamous cell carcinoma (HNSCC), but knowledge of the prevalence of and risk factors for oral HPV infection, especially cutaneous types in Iran, remains unknown. In a large retrospective study, the authors used a sensitive assay for the detection of α-, β-, and γ-HPVs in oral rinse samples of HNSCC and matched controls. They find that the α-HPV contribution to HNSCC in Iran is lower than global prevalence. High-risk α-HPVs or cutaneous β- and γ-HPVs were not associated with the HNSCC development. Besides, this study provides novel insights into the diversity of β- and γ-HPVs in different HNSCC anatomical subsites.

## INTRODUCTION

Head and neck squamous cell carcinoma (HNSCC) is a debilitating disease that involves multiple anatomical subsites that comprise the lip and oral cavity, pharynx, and larynx (1). Annually more than 850,000 people are diagnosed with HNSCC worldwide with 5%of all types of tumors leading to more than 450,000 HNSCC-related deaths (2). HNSCC was among the top 10 cancers by incidence worldwide in 2018 for which the established risk factors are tobacco products, excessive alcohol drinking, areca (betel) nut, UV light exposure, occupational exposures such as perchloroethylene and trichloroethylene, and infections like Epstein-Barr virus (EBV) and human papillomavirus (HPV) (2–5).

A broad spectrum of mucosal and cutaneous HPV types has been isolated from oral cavity samples (6–9). Certain mucosal high-risk HPV types are associated with the HNSCC development (10, 11), yet the contribution of nonmucosal HPVs comprising beta, gamma, mu, and nu HPV types is still unclear. Some studies have highlighted the link between nonalpha HPVs (including β1-HPV5) and γ-species 11 and 12 in the development of HNSCC (6, 8, 10). The epidemiology of HPV-related HNSCC varies and is highly dependent on tumor subsite and geographical area (12). The overall prevalence of HPV infection in HNSCC has been estimated around 25% to 32% based on large meta-analysis studies over the last 2 decades (13–15). The prevalence of HPV infection in patients with HNSCC ranges from 3% to 60% in different geographical regions of Iran (16, 17). In a meta-analysis conducted by Ndiaye et al. on 12,163 HNSCC patients from 44 countries, the pooled HPV DNA prevalence was 46% for oropharynx, 22% for larynx (including hypopharynx), and 24% for the oral cavity (13). HPV16 is known to be the most common type in a subset of HNSCC, constituting 80% to 100% of HPV DNA-positive oropharyngeal region (i.e., tonsils and base of the tongue) (18, 19). HPV infection is found in 20% to 60% of the oropharyngeal squamous cell carcinomas (OPSCCs) depending on the countries, with the highest rate in Western Europe and North America. It is now established that HPV is strongly linked to the risk of developing OPSCCs (20). Compared with the HPV-negative HNSCC, the HPV-related HNSCC is expected to have different risk factors, for instance, sexual behavior instead of tobacco and alcohol consumption (12).

There is a discrepancy in the HPV-attributable fraction of HNSCC among Iranian patients (16, 17, 21–24). Furthermore, the prevalence of HPV in HNSCC subsites and the determinants of α-, β-, and γ-HPVs types need further evaluation. The present study was a large case-control study exploring the association of HPV and HNSCC in Iranian patients. We assessed the association of HPV and subsites of head and neck cancer, including lip and oral cavity, pharynx, and larynx.

## RESULTS

**FIG 1:**
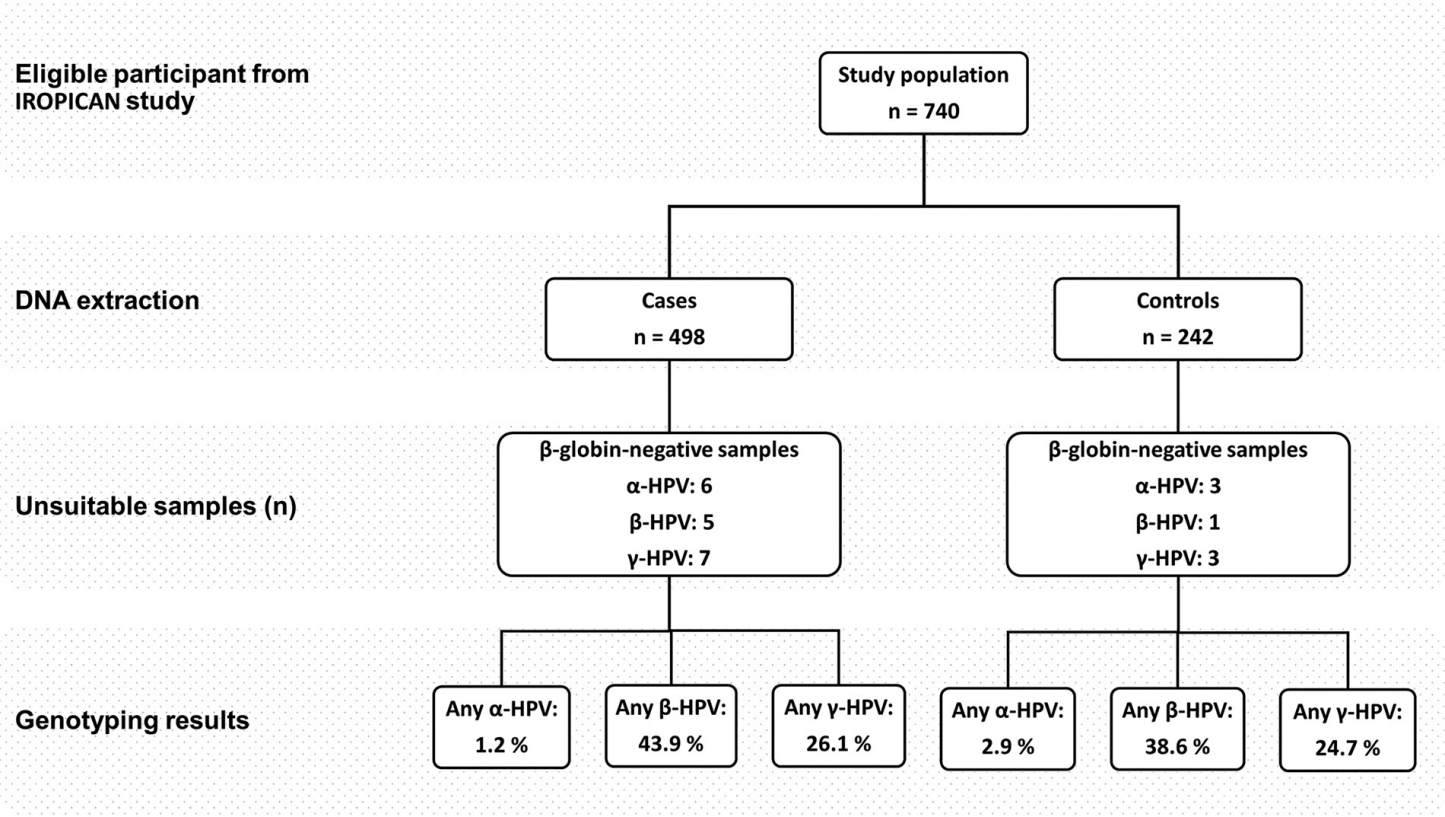
Flowchart of the study design and HPV prevalence in patients and controls.

A total of 498 HNSCC cases, of which 198 lip and oral cavity (International Classification of Diseases for Oncology [ICD-O]: C00-C08 and C14), 44 pharynx (32 nasopharynx, four oropharynx, and eight hypopharynx; ICD-O: C09-C11 and C13), 219 larynx (ICD-O: C32), and 37 other subsites of the head and neck (ICD-O: C12, C30, C31, and C76), and 242 controls from the IROPICAN study were included in the study. All β-globin negative samples were excluded from the final analysis (Fig. 1). Characteristics and available information for the study groups are presented in Table 1.

**TABLE 1:** Demographic and lifestyle characteristics and α-, β-, and γ-HPV status of 498 HNSCC cases and 242 matched controls

Characteristics	Controls	HNCC cases
Gender, No. (%)		
Male	195 (80.6)	377 (75.7)
Female	47 (19.4)	121 (24.3)
Mean age ± SD	54.3 ± 11.1	57.4 ± 12.2
Place of residence, No. (%)		
Capital city	187 (77.3)	332 (66.7)
Noncapital city	42 (17.4)	115 (23.1)
missing	13 (5.4)	51 (10.2)
Tobacco user, No. (%)		
Yes	140 (57.9)	283 (56.9)
No	102 (42.2)	215 (43.2)
Opium user, No. (%)		
Yes	89 (36.8)	183 (36.8)
No	153 (63.2)	315 (63.3)
Any α-HPV infection, No. (%)^*a*^		
Positive	7 (2.9)	6 (1.2)
Negative	232 (97.1)	486 (98.8)
Any β-HPV infection, No. (%)^*a*^		
Positive	93 (38.6)	216 (43.9)
Negative	148 (61.4)	277 (56.2)
Any γ-HPV infection, No. (%)^*a*^		
Positive	59 (24.7)	128 (26.1)
Negative	180 (75.3)	363 (73.9)
Anatomic subsites, No. (%)		
Lip and Oral cavity	-^*b*^	198 (39.8)
Pharynx	-	44 (8.8)
Nasopharynx	-	32 (72.7)
Oropharynx	-	4 (9.1)
Hypopharynx	-	8 (18.2)
Larynx	-	219 (44.0)
Other	-	37 (7.4)

a Six, 5, and 7 patients and 3, 1, and 3 controls samples had negative ß-globin PCR assay results, respectively, for α-, β-, and γ-HPV genotyping which are excluded from the final analysis.

b Not relevant for control group.

### α-, β-, and γ-HPV DNA positivity prevalence.

Six out of 492 (1.2%) cases and seven out of 239 (2.9%) controls were positive for any α-HPVs. Characteristics of the 13 α-HPV-positive participants are reported in Table S1. Nine types of 21 types (HPV6, HPV11, HPV16, HPV18, HPV53, HPV56, HPV58, HPV70, and HPV82) were detected in the participants (Table S1). HPV16 (*n* = 4; 0.8%) was the most prevalent α-HPV type among the HNSCC patients, and controls (*n* = 2; 0.8%).

The prevalence of any β-HPV was 43.8% (*n* = 216) among the patients and 38.6% (*n* = 93) among the controls. The lip and oral cavity showed the highest prevalence of β-HPV DNA (90/198, 45.6%), followed by larynx (96/215, 44.7%), pharynx (10/43, 23.3%), and other anatomical subsites (20/37, 54.1%) (Table 2).

**TABLE 2:** Prevalence of type-specific β-HPV infection in oral rinse samples of HNSCC cases and matched controls

	Controls (*N* = 241)	HNSCC cases				
		Lip and oral cavity (*N* = 198)	Pharynx (*N* = 43)	Larynx (*N* = 215)	Other subsites (*N* = 37)	Any HNSCC (*N* = 493)
β-HPV type^*a*^	No. (%)	No. (%)	No. (%)	No. (%)	No. (%)	No. (%)
Any β-Positive	93 (38.6)	90 (45.5)	10 (23.3)	96 (44.7)	20 (54.1)	216 (43.8)
Single type	41 (17.0)	45 (22.7)	7 (16.3)	41 (19.1)	7 (18.9)	100 (20.3)
Multiple type	52 (21.6)	45 (22.7)	3 (7.0)	55 (25.6)	13 (35.1)	116 (23.5)
Any β1	53 (22.0)	59 (29.8)	5 (11.6)	62 (28.8)	10 (27.0)	136 (27.6)
HPV5	14 (5.8)	15 (7.6)	2 (4.7)	11 (5.1)	2 (5.4)	30 (6.1)
HPV8	4 (1.7)	6 (3.0)	0	5 (2.3)	2 (5.4)	13 (2.6)
HPV12	8 (3.3)	14 (7.1)	0	8 (3.7)	0	22 (4.4)
HPV14	4 (1.7)	8 (4.0)	1 (2.3)	6 (2.8)	2 (5.4)	17 (3.5)
HPV19	8 (3.3)	10 (5.1)	0	8 (3.7)	2 (5.4)	20 (4.1)
HPV20	5 (2.1)	2 (1.0)	0	4 (1.9)	3 (8.1)	9 (1.8)
HPV21	11 (4.6)	8 (4.0)	1 (2.3)	16 (7.4)	3 (8.1)	28 (5.7)
HPV24	10 (4.2)	12 (6.1)	0	14 (6.5)	4 (10.8)	30 (6.1)
HPV25	2 (0.8)	0	0	1 (0.5)	0	1 (0.2)
HPV36	3 (1.2)	1 (0.5)	0	1 (0.5)	1 (2.7)	3 (0.6)
HPV47	6 (2.5)	8 (4.0)	1 (2.3)	8 (3.7)	4 (10.8)	21 (4.3)
HPV93	3 (1.2)	3 (1.5)	0	5 (2.3)	1 (2.7)	9 (1.8)
HPV98	6 (2.5)	4 (2.0)	0	3 (1.4)	2 (5.4)	9 (1.8)
HPV99	9 (3.7)	4 (2.0)	0	5 (2.3)	4 (10.8)	13 (2.6)
HPV105	9 (3.7)	12 (6.1)	2 (4.7)	4 (1.9)	1 (2.7)	19 (3.9)
HPV118	1 (0.4)	2 (1.0)	0	1 (0.5)	1 (2.7)	4 (0.8)
HPV124	19 (7.9)	8 (4.0)	0	13 (6.1)	2 (5.4)	23 (4.7)
HPV143	2 (0.8)	1 (0.5)	0	1 (0.5)	0	2 (0.4)
Any β2	73 (30.3)	57 (28.8)	6 (12.8)	73 (34.0)	17 (46.0)	153 (31.0)
HPV9	9 (3.7)	6 (3.0)	1 (2.3)	5 (2.3)	2 (5.4)	14 (2.8)
HPV15	5 (2.1)	1 (0.5)	0	4 (1.9)	1 (2.7)	6 (1.2)
HPV17	5 (2.1)	0	0	0	0	0
HPV22	8 (3.3)	6 (3.0)	0	4 (1.9)	0	10 (2.0)
HPV23	20 (8.3)	16 (8.1)	2 (4.7)	21 (9.8)	5 (13.5)	44 (8.9)
HPV37	2 (0.8)	1 (0.5)	0	0	2 (5.4)	3 (0.6)
HPV38	22 (9.1)	19 (9.6)	1 (2.3)	16 (7.4)	3 (8.1)	39 (7.9)
HPV80	0	0	0	3 (1.4)	0	3 (0.6)
HPV100	4 (1.7)	10 (5.1)	0	10 (4.7)	1 (2.7)	21 (4.3)
HPV104	1 (0.4)	1 (0.5)	0	6 (2.8)	1 (2.7)	8 (1.6)
HPV107	4 (1.7)	1 (0.5)	0	6 (2.8)	1 (2.7)	8 (1.6)
HPV110	3 (1.2)	3 (1.5)	0	7 (3.3)	0	10 (2.0)
HPV111	13 (5.4)	3 (1.5)	2 (4.7)	5 (2.3)	4 (10.8)	14 (2.8)
HPV113	1 (0.4)	0	0	2 (0.9)	0	2 (0.4)
HPV120	7 (2.9)	5 (2.5)	1 (2.3)	5 (2.3)	4 (10.8)	15 (3.0)
HPV122	8 (3.3)	2 (1.0)	1 (2.3)	10 (4.7)	2 (5.4)	15 (3.0)
HPV145	14 (5.8)	10 (5.1)	0	13 (6.1)	5 (13.5)	28 (5.7)
HPV151	2 (0.8)	1 (0.5)	0	4 (1.9)	3 (8.1)	8 (1.6)
HPV159	10 (4.2)	5 (2.5)	0	15 (7.0)	2 (5.4)	22 (4.5)
HPV174	3 (1.2)	7 (3.5)	0	10 (4.7)	3 (8.1)	20 (4.1)
Any β3	20 (8.3)	19 (9.6)	2 (4.7)	24 (11.2)	6 (16.2)	51 (10.3)
HPV49	11 (4.6)	7 (3.5)	1 (2.3)	14 (6.5)	3 (8.1)	25 (6.0)
HPV75	3 (1.2)	3 (1.5)	0	6 (2.8)	2 (5.4)	11 (2.2)
HPV76	9 (3.7)	11 (5.6)	0	10 (4.7)	2 (5.4)	23 (4.7)
HPV115	2 (0.8)	3 (1.5)	1 (2.3)	1 (0.5)	0	5 (1.0)
Any β4	3 (1.2)	2 (1.0)	0	1 (0.5)	2 (5.4)	5 (1.0)
HPV92	3 (1.2)	2 (1.0)	0	1 (0.5)	2 (5.4)	5 (1.0)
Any β5	5 (2.1)	2 (1.0)	0	3 (1.4)	1 (2.7)	6 (1.2)
HPV96	4 (1.7)	1 (0.5)	0	2 (1.0)	1 (2.7)	4 (0.8)
HPV150	1 (0.4)	1 (0.5)	0	1 (0.5)	0	2 (0.4)

a HPV152 was not detected in saliva samples of cases and controls.

HPVs from β-HPV-2, -1, -3, -5, and -4 species were the most abundant in patients and controls (Table 2).

The most frequent β-HPV types detected in HNSCC patients were HPV23 (8.9%), HPV38 (7.9%), HPV24 (6.1%), HPV5 (6.1%), and HPV49 (6.0%), while the most frequent β-HPV types in controls were HPV38 (9.1%), HPV23 (8.3%), HPV124 (7.9%), HPV145 (5.8%), and HPV111 (5.4%). Multiple β-HPV infections were found in 23.5% (116 out of 216 β-HPV-positive samples) and 21.6% (52 out of 93 β-HPV-positive samples) of the patients and controls, respectively (Table 2).

The prevalence of any γ-HPV among the cases and controls were 26.1% (*n* = 128) and 24.9% (*n* = 59), respectively. The larynx subsite contained most of the γ-HPV positive samples (65/214, 30.4%), followed by the lip and oral cavity (49/197, 24.9%), other subsites (7/37, 19.0%), and pharynx (7/43, 16.3%) (Table 3). For γ-HPVs, 38 were detected out of the 52 screened types. The corresponding γ-HPV-10, -7, -1, SD, -4, -19, -23, and -24 species (by a decreasing order of frequency) in the patients and γ-HPV-7, -10, -4, -24, -12, SD, and -8 species in the controls had the highest prevalence (Table 3). The most common γ-HPV types in the patient samples were SD2 (3.7%), HPV156 (3.3%), HPV121 (2.9%), HPV161 (2.7%), HPV134 (2.4%), and HPV130 (2.4%), while HPV134 (5.4%), HPV156 (4.2%), HPV130 (3.4%), HPV133 (3.0%), and HPV SD2 (3.0%) were the most prevalent γ-HPV types found in the control samples. Multiple γ-HPV infections were detected in 10.2% (*n* = 50) of the patients and 9.2% (*n* = 22) of the controls (Table 3).

**TABLE 3:** Prevalence of type-specific γ-HPV infection in oral rinse samples of HNSCC cases and matched controls

γ-HPV type^*a*^	Controls (*N* = 239)	HNSCC cases				
		Lip and oral cavity (*N* = 197)	Pharynx (*N* = 43)	Larynx (*N* = 214)	Other subsites (*N* = 37)	Any HNSCC (*N* = 491)
Any γ-positive	59 (24.7)	49 (24.9)	7 (16.3)	65 (30.4)	7 (18.92)	128 (26.1)
Single type	37 (15.5)	31 (15.7)	4 (9.3)	37 (17.3)	6 (16.22)	78 (15.9)
Multiple type γ	22 (9.2)	18 (9.1)	3 (7.0)	28 (13.1)	1 (2.70)	50 (10.2)
γ-species1	3 (1.3)	10 (5.1)	1 (2.3)	10 (4.7)	0	21 (4.3)
HPV4	2 (0.8)	6 (3.1)	0	5 (2.3)	0	11 (2.2)
HPV65	1 (0.4)	2 (1.0)	1 (2.3)	2 (0.9)	0	5 (1.0)
HPV95	0	3 (1.5)	0	3 (1.4)	0	6 (1.2)
γ-species 4						
HPV156	10 (4.2)	5 (2.5)	0	11 (5.1)	0	16 (3.3)
γ-species 5						
HPV60	0	2 (1.0)	0	1 (0.5)	0	3 (0.6)
γ-species 6						
HPV108	2 (0.8)	0	0	1 (0.5)	0	1 (0.2)
γ-species 7	17 (7.1)	13 (6.6)	2 (4.7)	12 (5.6)	1 (2.7)	28 (5.7)
HPV123	0	3 (1.5)	0	4 (1.9)	0	7 (1.4)
HPV134	13 (5.4	8 (4.1)	1 (2.3)	3 (1.4)	0	12 (2.4)
HPV149	4 (1.9)	1 (0.5)	0	4 (1.9)	1 (2.7)	6 (1.2)
HPV170	0	1 (0.5)	1 (2.3)	1 (0.5)	0	3 (0.6)
γ-species 8	5 (2.1)	2 (1.0)	0	5 (2.3)	0	7 (1.4)
HPV112	0	1 (0.5)	0	0	0	1 (0.2)
HPV164	0	1 (0.5)	0	3 (1.4)	0	4 (0.8)
HPV168	5 (2.1)	0	0	2 (0.9)	0	2 (0.4)
γ-species 9						
HPV129	0	1 (0.5)	0	1 (0.5)	0	2 (0.4)
γ-species10	17 (7.1)	10 (5.1)	4 (9.3)	16 (7.5)	1 (2.7)	31 (6.3)
HPV121	3 (1.3)	4 (2.0)	2 (4.7)	7 (3.3)	1 (2.7)	14 (2.9)
HPV130	8 (3.4)	5 (2.5)	0	7 (3.3)	0	12 (2.4)
HPV133	7 (2.9)	1 (0.5)	2 (4.7)	7 (3.3)	1 (2.7)	11 (2.2)
HPV180	3 (1.3)	2 (1.0)	2 (4.7)	4 (1.9)	1 (2.7)	9 (1.8)
γ-species 11	2 (0.9)	2 (1.0)	0	2 (0.9)	1 (2.7)	5 (1.0)
HPV126	1 (0.4)	0	0	0	1 (2.7)	1 (0.2)
HPV169	1 (0.4)	2 (1.0)	0	2 (0.9)	0	4 (0.8)
HPV202	1 (0.4)	0	0	0	0	0
γ-species 12	7 (2.9)	3 (1.5)	0	4 (1.9)	1 (2.7)	8 (1.6)
HPV127	1 (0.4)	2 (1.0)	0	1 (0.5)	0	3 (0.6)
HPV132	4 (1.7)	1 (0.5)	0	4 (1.9)	1 (2.7)	6 (1.2)
HPV148	1 (0.4)	0	0	0	0	0
HPV199	1 (0.4)	0	0	0	0	0
γ-species 13						
HPV128	4 (1.7)	3 (1.5)	0	6 (2.8)	0	9 (1.8)
γ-species 15						
HPV179	1 (0.4)	1 (0.5)	0	7 (3.3)	1 (2.7)	9 (1.8)
γ-species 19	3 (1.3)	1 (0.5)	0	12 (5.6)	1 (2.7)	14 (2.9)
HPV161	2 (0.84)	0	0	12 (5.61)	1 (2.70)	13 (2.7)
HPV162	1 (0.42)	1 (0.51)	0	0	0	1 (0.2)
HPV166	0	1 (0.51)	0	0	0	1 (0.2)
γ-species 20						
HPV163	0	0	0	1 (0.5)	0	1 (0.2)
γ-species 21						
HPV167	2 (0.8)	3 (1.5)	1 (2.3)	1 (0.5)	0	5 (1.0)
γ-species 22						
HPV172	2 (0.8)	1 (0.5)	0	4 (1.9)	0	5 (1.02)
γ-species 23						
HPV175	4 (1.7)	2 (1.0)	0	7 (3.3)	1 (2.7)	10 (2.0)
γ-species 24	8 (3.4)	6 (3.1)	0	4 (1.9)	0	10 (2.0)
HPV178	3 (1.3)	3 (1.5)	0	2 (0.9)	0	5 (1.0)
HPV197	5 (2.1)	3 (1.5)	0	2 (0.9)	0	5 (1.0)
γ-species 27	0					2 (0.4)
HPV201	0	1 (0.5)	0	1 (0.5)	0	2 (0.4)
SD2	7 (2.9)	6 (3.1)	1 (2.33)	9 (4.2)	2 (5.4)	18 (3.7)

a The following γ-HPV types were not detected in the oral rinse samples of subjects: HPV48, HPV119, HPV50, HPV131, HPV88, HPV165, HPV101, HPV171, HPV103, HPV173, HPV109, HPV184, HPV116, and HPV200.

Based on the multivariate model, we found no associations between the risk of HNSCC and α-HPV (ORs; 0.4, 95% CI = 0.1 to 1.2), β-HPV (ORs; 1.2, 95% CI = 0.9 to 1.6), and γ-HPV (ORs: 1.0, 95% CI = 0.7 to 1.5). There was no significant difference in the frequency of individual α, β, and γ types between the cases and controls except for HPV134 (*P* = 0.041) and HPV168 (*P* = 0.048) (Table 4). We observed no associations between any HPV types and cancers of pharynx subsites (Table 5).

**TABLE 4:** Association between α-, β-, and γ-HPV infections and HNSCC

HPV positivity	Crude OR (95% CI)	*P* value	Adjusted OR^*a*^ (95% CI)	*P* value^*b*^
Any α-HPV				
HPV−	1		1	
HPV+	0.4 (0.1 to 1.2)	0.112	0.40 (0.1 to 1.2)	0.111
Any β-HPV				
HPV−	1		1	
HPV+	1.2 (0.9 to 1.7)	0.179	1.18 (0.9 to 1.6)	0.299
Types associated with disease				
HPV100	2.6 (0.9 to 7.8)	*0.079*	2.75 (0.9 to 8.2)	*0.070*
HPV111	0.5 (0.2 to 1.1)	*0.089*	0.5 (0.2 to 1.2)	0.116
HPV124	0.6 (0.3 to 1.1)	*0.081*	0.5 (0.3 to 1.0)	*0.061*
HPV174	3.4 (0.98 to 11.4)	*0.053*	3.1 (0.9 to 10.6)	*0.073*
Any γ-HPV				
HPV−	1		1	
HPV+	1.1 (0.8 to 1.5)	0.688	1.0 (0.7 to 1.5)	0.830
Types associated with disease				
HPV95	zero HPV95 in controls	-^*c*^	-	*-*
HPV123	zero HPV95 in controls	-	-	*-*
HPV134	0.4 (0.2 to 0.96)	*0.042*	0.42 (0.2 to 0.96)	**0.041**
HPV168	0.19 (0.0 to 0.99)	*0.049*	0.18 (0.0 to 0.98)	**0.048**

a ORs were adjusted for age (10-year intervals), gender, place of residence, tobacco use, opium use, and socioeconomic status.

b Italic *p* values indicate positive trends. Bold *p* values indicate a significant association.

c Due to zero number, it was not possible to estimate ORs and 95% CIs.

**TABLE 5:** Association of positivity of HPV infections and Pharynx SCC by anatomic sites

	Pharynx			Nasopharynx (32)		Oropharynx (4)		Hypopharynx (8)	
HPV positivity	Case/control	Crude OR (95%CI)	Adjusted OR^*a*^ (95% CI)	Case/control	Adjusted OR^*a*^ (95% CI)	Case/control	Adjusted OR^*a*^ (95% CI)	Case/control	Adjusted OR^*a*^ (95% CI)
Any α-HPV									
HPV−	44/232	-^*b*^	-	32/232	-	4/232	-	8/232	-
HPV+	0/7	-	-	0/7	-	0/7	-	0/7	-
Any β-HPV									
HPV−	33/148	1	1	28/148	1	1/148	1	4/148	1
HPV+	10/93	0.5 (0.2 to 1.0), *P* = 0.058	0.6 (0.3 to 1.2), *P* = 0.143	4/93	0.3 (0.1 to 0.8), *P* = 0.018	2/93	3.6 (0.3 to 45.4), *P* = 0.324	4/93	1.9 (0.4 to 8.4), *P* = 0.400
Any γ-HPV									
HPV−	36/180	1	1	26/180	1	3/180	-	7/180	1
HPV+	7/59	0.6 (0.3 to 1.4), *P* = 0.235	0.61 (0.3 to 1.5), *P* = 0.282	6/59	0.77 (0.3 to 2.1), *P* = 0.610	0/59	-	1/59	0.4 (0.0 to 3.6), *P* = 0.419

a ORs were adjusted for age (10-year intervals), gender, place of residence, tobacco use, and opium use.

b Due to zero number, it was not possible to estimate ORs and 95% CIs.

## DISCUSSION

Recent progress has highlighted the importance of HPV infections in the HNSCC pathogenesis from which high-risk mucosal HPV types are considered causal factors, especially in the oropharyngeal cancer development (19, 25–27). Compared with the alpha genus, the contribution of beta and gamma genera solely or in combination with other risk factors to the carcinogenesis of HNSCC needs to be explored further. Here, we studied the contribution of HPV infections to the HNSCC development in a multicenter case-control study in Iran. The presence of HPV DNA from 119 types was determined using highly sensitive type-specific bead-based multiplex genotyping assays developed at IARC (28–30).

In this study, a small fraction of HNSCC was positive for α-HPV DNA, lower than the previous estimates from Iran that mainly involved oral cavity and larynx fresh frozen and formalin-fixed paraffin-embedded (FFPE) samples (16, 31). Furthermore, using the FFPE samples collected from the northwest of Iran, 43% of HR-HPV types were ascribed to the oral cavity, hypopharynx, and laryngeal cancer patients in which HPV18 and HPV16 were the most common types (24). In addition, 4.3% of the saliva samples from the control group were HR-HPV positive (24) while the prevalence was less than 3% in our control group in line with other studies (19, 25, 32), HPV16 was the most common type among our 13 α-HPV positive participants.

Previous findings are in favor of an increased risk of oropharyngeal cancers with the HPV infection (10, 33). However, in this study, we did not find high-risk HPV types among four oropharyngeal samples. While in one unpublished study of ours, using GP5+ and GP6+ primers that amplify a broad spectrum of HPV genotypes, we found 17 out of 136 FFPE oropharynx tumor samples were HPV positive.

We also provided additional data regarding the β- and γ-HPV prevalence in the HNSCC cases and matched controls. According to our results, 43.8% of the patients and 38.6% of the controls were β-HPV positive, in a fair agreement with a report of 41.8% positivity for any β-HPV in the samples from the oral region of the head and neck cancer patients and controls (8). We detected a broad diversity of β-HPV types among 46 tested types. Such diversity has been found in healthy men’s anogenital and oral anatomic sites in a target population from Brazil and Russia (30, 34). In line with our findings, in the study of Paolini et al. on oral rinse samples from healthy controls and HNC cases, 51% of the patients and 23% of the controls were β-HPV positive (35).

In a nested case-control study by Agalliu et al., 62.1% of the HNSCC patients were positive for any β-HPV (mainly any β1 and β2). Any β-HPV positive cases had an increased risk of HNSCC incidence by two times more than the controls; the same association occurred for the types β1-HPV5 and β2-HPV38. In addition, the β1-HPV5 type increased the risk of oropharyngeal, oral cavity, and laryngeal cancers (10). In the current study, no association was observed between the positivity for the β-HPV infections and HNSCC even after adjusting for the potential confounders. Our data did not establish any causality for the β-HPV and HNSCC risk and the obtained β-HPV may be a subset of transient infections driven from the skin. This may be attributed to the main objective of IROPICAN study which was not to exclusively explore the association of HPV infections with the risk of HNSCC (36). It would be interesting to study the prevalence of β-HPVs at different cutaneous anatomical regions of patients to better understand the transmission routes of β-HPV infection.

The higher prevalence of β1- and -2 HPV species in our findings are inconsistent with the previous reports (8, 10). According to our results, HPV23 and HPV38 were the most common types in the oral rinse samples of the patients and controls. Other studies have reported HPV23 as the most prevalent type detected in the skin of immunosuppressed and immunocompetent individuals, and also the most common type found in the oral cavity of healthy people (6, 37). Further, HPV38 is involved in the development of nonmelanoma skin cancer (38). Our results did not confirm the previous studies that reported HPV5 as the most prevalent type and HPV38 as the second most common type (8, 10). Similar to other studies (34), this study could not distinguish new or persistent HPV infections. As expected in the rinse samples, multiple β-HPV infections were also found in the patients and controls.

Similar to β genus, we did not find any association between the γ-HPV positivity and HNSCC risk. In this study, γ7-HPV134 and γ8-HPV168 were found to be statistically significantly associated with the HNSCC risk. The possible role of these cutaneous HPV types in the development of head and neck cancer needs further evaluation. The γ-HPV genus is a ubiquitous and continuously growing HPV genus found in both cutaneous and mucosal sites (39, 40). Information on their prevalence and consequences in the head and neck is limited. Our results showed that the γ genus was the second prevalent HPV genus in the oral gargle samples of HNSCC patients, confirming previous reports that γ-HPVs are frequently present at mucosal sites (41). γ-HPV-10 and -7 were the most common γ-species among our patients similar to those observed by Agalliu et al. (10). Moreover, the Agalliu et al. found a direct association between any γ11-HPV, and any γ12-HPV infections and the risk of HNSCC (10).

We are aware that a number of limitations may have influenced our results. Firstly, patients with tumors in oral, oropharyngeal, and larynx/pharynx sites may not have done appropriate specimen collection containing the epithelial cells; this may result in a small amount of HPV containing mucus and debris from the exfoliated cells. Secondly, an extremely rare number of oropharyngeal cancer patients in the current study caused inefficient power to detect HPV infection. It is noteworthy that in general, less than 1% of head and neck cancers in Iran are oropharyngeal ones. Hence, we suggest a pooled analysis of oropharyngeal cancers from the WHO Eastern Mediterranean Regional Office (EMRO) region or the International Head and Neck Cancer Epidemiology (INHANCE) consortium.

In conclusion, our data were not suggestive of an HPV-related etiology in the HNSCC patients in Iran. However, this study provides novel insights into the diversity of β-, and γ-HPVs in different HNSCC anatomical subsites. To better understand the natural history and transmission dynamics of β- and γ-HPV in patients versus controls in the development of HNSCC, studying the HPV status in the anogenital and anal specimens is highly recommended.

## MATERIALS AND METHODS

### Study design and population.

Oral rinse specimens were originated from the Iran Opium and Cancer (IROPICAN) study, which was conducted in 10 different provinces of Iran among HNSCC cases and sex- and age-matched controls during April 2016 and April 2019. Detailed information on the study method and design has been described elsewhere (42). Briefly, we obtained oral rinses from 498 pathologically confirmed HNSCC cases and 242 hospital controls from the IROPICAN study. Hospital visitor controls were determined as valid and reliable controls versus neighborhood and disease controls (43). In addition to oral rinse collection, a comprehensive questionnaire including the other risk factors was also filled out through a face-to-face interview. Detailed questions were about opium, tobacco use (cigarette and water-pipe smoking), oral health, and alcohol drinking. Definitions and assessment of opium exposure have been described in detail elsewhere (42). In this study, the use of main opium types, including raw opium (teriak), refined opium (shireh), and opium dross (sukhteh) was considered among opium users.

### Oral specimen collection.

Patients and controls were instructed to rinse their mouth vigorously with 10 mL of mouthwash (normal saline solution) for 60 s. The patients were asked to do this before surgery to restrict the presence of blood cells in the oral rinse, then spit it into RNase/DNase-free 50 mL conical centrifuge tubes. The samples were then centrifuged for 10 min, and the oral cell pellets were resuspended in 1 mL of remaining saline solution and stored at −80°C.

### HPV DNA genotyping.

Oral rinse samples were shipped to the International Agency for Research on Cancer (Lyon, France) for DNA extraction and HPV DNA genotyping. Samples were centrifuged at 4,000 g for 5 min for pelleting the oral cell and washed with phosphate-buffered saline. Nucleic acid extraction was performed using the EZ1 DNA Tissue kit. Briefly, cell pellets were then resuspended in 180 μL of G2 buffer (Qiagen, Hilden, Germany) and 20 μL of proteinase K (Qiagen) and incubated overnight at 56°C under agitation at 750 rpm in a Thermomixer Comfort instrument (Eppendorf, Hamburg, Germany). DNA was then extracted using the EZ1 instrument (Qiagen), according to the manufacturer’s instructions, eluted in a final volume of 50 μL, and stored at 20°C until analysis. The samples were then tested for the presence of α-, β-, and γ-HPV types using multiplex bead-based assays that combine multiplex PCRs and Luminex technology (Luminex Corp., Austin, TX, USA), as described elsewhere (44, 45).

The multiplex type-specific PCR method uses specific primers for the detection of 21 high-risk α-HPV types (6, 11, 16, 18, 26, 31, 33, 35, 39, 45, 51, 52, 53, 56, 58, 59, 66, 68, 70, 73, 82); 46 β-HPVs from β-species 1 (5, 8, 12, 14, 19, 20, 21, 24, 25, 36, 47, 93, 98, 99, 105, 118, 124, 143, and 152), β-species 2 (9, 15, 17, 22, 23, 37, 38, 80, 100, 104, 107, 110, 111, 113, 120, 122, 145, 151, 159, and 174), β-species 3 (49, 75, 76, and 115), β-species 4 (92), and β-species 5 (96 and 150); and 52 γ-HPVs from γ-species 1 (4, 65, 95, and 173), γ-species 2 (48 and 200), γ-species 3 (50), γ-species 4 (156), γ-species 5 (60, 88), γ-species 6 (101, 103, and 108), γ-species 7 (109, 123, 134, 149, and 170), γ-species 8 (112, 119, 164, and 168), γ-species 9 (116 and 129), γ-species 10 (121, 130, 133, and 180), γ-species 11 (126, 169, 171, and 202), γ-species 12 (127, 132, 148, 165, and 199), γ-species 13 (128), γ-species 14 (131), γ-species 15 (179), γ-species 18 (156), γ-species 19 (161, 162, and 166), γ-species 20 (163), γ-species 21 (167), γ-species 22 (172), γ-species 23 (175), γ-species 24 (178 and 197), γ-species 25 (184), and γ-species 27 (201), and SD2 (46). Amplification of β-globin DNA in the samples was considered a positive control for the quality of the template DNA in the samples. Negative samples for β-globin DNA were excluded from the final analysis.

### Statistical analysis.

All statistical analyses were conducted using Stata, version 14 (Stata Corp, College Station, TX). Frequencies and percentages were calculated for categorical variables. We used a logistic regression model and adjusted it for the study centers, age, sex, place of residence, tobacco use, opium use, and socioeconomic status to estimate the association between genotypes of α-, β-, and γ-HPVs and risk of HNSCC. We chose the covariates of models if the type I error was less than *P* = 0.2 (47). Moreover, we used the stepwise likelihood ratio tests to identify any other potential confounders. We actively sought the collinearity of the covariates; in case of any collinearity, we omitted the covariates or integer them, e.g., cigarette smoking and water-pipe smoking were colinear, and we generated a tobacco variable. Further, models were stratified by pharynx cancer subsites as nasopharynx, oropharynx, and hypopharynx.

### Data availability.

The data set used and analyzed during study are available from the corresponding authors on reasonable request.

## References

[1] Lo Nigro C, Denaro N, Merlotti A, Merlano M. 2017. Head and neck cancer: improving outcomes with a multidisciplinary approach. Cancer Manag Res 9:363–371. doi:10.2147/CMAR.S115761. 28860859 PMC5571817

[2] Bray F, Ferlay J, Soerjomataram I, Siegel RL, Torre LA, Jemal A. 2018. Global cancer statistics 2018: GLOBOCAN estimates of incidence and mortality worldwide for 36 cancers in 185 countries. CA Cancer J Clin 68:394–424. doi:10.3322/caac.21492. 30207593

[3] Stenson KM. 2020. Epidemiology and risk factors for head and neck cancer. https://www.uptodate.com/contents/epidemiology-and-risk-factors-for-head-and-neck-cancer.

[4] Chow LQM. 2020. Head and neck cancer. N Engl J Med 382:60–72. doi:10.1056/NEJMra1715715. 31893516

[5] Carton M, Barul C, Menvielle G, Cyr D, Sanchez M, Pilorget C, Trétarre B, Stücker I, Luce D, ICARE Study Group. 2017. Occupational exposure to solvents and risk of head and neck cancer in women: a population-based case-control study in France. BMJ Open 7:e012833. doi:10.1136/bmjopen-2016-012833. PMC5223686 28069619

[6] Bottalico D, Chen Z, Dunne A, Ostoloza J, McKinney S, Sun C, Schlecht NF, Fatahzadeh M, Herrero R, Schiffman M, Burk RD. 2011. The oral cavity contains abundant known and novel human papillomaviruses from the betapapillomavirus and gammapapillomavirus genera. J Infect Dis 204:787–792. doi:10.1093/infdis/jir383. 21844305 PMC3156102

[7] Garbuglia AR. 2014. Human papillomavirus in head and neck cancer. Cancers (Basel) 6:1705–1726. doi:10.3390/cancers6031705. 25256828 PMC4190563

[8] Sabol I, Smahelova J, Klozar J, Mravak-Stipetic M, Gheit T, Tommasino M, Grce M, Tachezy R. 2016. Beta-HPV types in patients with head and neck pathology and in healthy subjects. J Clin Virol 82:159–165. doi:10.1016/j.jcv.2016.07.019. 27500365

[9] Wong MCS, Vlantis AC, Liang M, Wong PY, Ho WCS, Boon SS, Sze RKH, Leung C, Chan PKS, Chen Z. 2018. Prevalence and epidemiologic profile of oral infection with alpha, beta, and gamma papillomaviruses in an Asian Chinese population. J Infect Dis 218:388–397. doi:10.1093/infdis/jiy160. 29982800 PMC6049037

[10] Agalliu I, Gapstur S, Chen Z, Wang T, Anderson RL, Teras L, Kreimer AR, Hayes RB, Freedman ND, Burk RD. 2016. Associations of oral α-, β-, and γ-human papillomavirus types with risk of incident head and neck cancer. JAMA Oncol 2:599–606. doi:10.1001/jamaoncol.2015.5504. 26794505 PMC4956584

[11] Fakhry C, Psyrri A, Chaturvedhi A. 2014. HPV and head and neck cancers: state-of-the-science. Oral Oncol 50:353–355. doi:10.1016/j.oraloncology.2014.03.010. 24726207

[12] Wagner S, Sharma SJ, Wuerdemann N, Knuth J, Reder H, Wittekindt C, Klussmann JP. 2017. Human papillomavirus-related head and neck cancer. Oncol Res Treat 40:334–340. doi:10.1159/000477252. 28521311

[13] Ndiaye C, Mena M, Alemany L, Arbyn M, Castellsagué X, Laporte L, Bosch FX, de Sanjosé S, Trottier H. 2014. HPV DNA, E6/E7 mRNA, and p16INK4a detection in head and neck cancers: a systematic review and meta-analysis. Lancet Oncol 15:1319–1331. doi:10.1016/S1470-2045(14)70471-1. 25439690

[14] Kreimer AR, Clifford GM, Boyle P, Franceschi S. 2005. Human papillomavirus types in head and neck squamous cell carcinomas worldwide: a systematic review. Cancer Epidemiol Biomarkers Prev 14:467–475. doi:10.1158/1055-9965.EPI-04-0551. 15734974

[15] Guo L, Yang F, Yin Y, Liu S, Li P, Zhang X, Chen D, Liu Y, Wang J, Wang K, Zhu Y, Lv Q, Wang X, Sun X. 2018. Prevalence of human papillomavirus type-16 in head and neck cancer among the Chinese population: a meta-analysis. Front Oncol 8:619. doi:10.3389/fonc.2018.00619. 30619756 PMC6299118

[16] Karbalaie Niya MH, Safarnezhad Tameshkel F, Panahi M, Bokharaei Salim F, Monavari SHR, Keyvani H. 2017. Human papillomavirus investigation in head and neck squamous cell carcinoma: initial report from the low risk HPV types associations. Asian Pac J Cancer Prev 18:2573–2579. doi:10.22034/APJCP.2017.18.9.2573. 28952562 PMC5720669

[17] Jalilvand S, Shoja Z, Hamkar R. 2014. Human papillomavirus burden in different cancers in Iran: a systematic assessment. Asian Pac J Cancer Prev 15:7029–7035. doi:10.7314/apjcp.2014.15.17.7029. 25227786

[18] Gillison ML, Castellsagué X, Chaturvedi A, Goodman MT, Snijders P, Tommasino M, Arbyn M, Franceschi S. 2014. Eurogin Roadmap: comparative epidemiology of HPV infection and associated cancers of the head and neck and cervix. Int J Cancer 134:497–507. doi:10.1002/ijc.28201. 23568556

[19] Gheit T, Anantharaman D, Holzinger D, Alemany L, Tous S, Lucas E, Prabhu PR, Pawlita M, Ridder R, Rehm S, Bogers J, Maffini F, Chiocca S, Lloveras B, Kumar RV, Somanathan T, de Sanjosé S, Castellsagué X, Arbyn M, Brennan P, Sankaranarayanan R, Pillai MR, Gangane N, Tommasino M, HPV-AHEAD study group. 2017. Role of mucosal high-risk human papillomavirus types in head and neck cancers in central India. Int J Cancer 141:143–151. doi:10.1002/ijc.30712. 28369859

[20] Augustin JG, Lepine C, Morini A, Brunet A, Veyer D, Brochard C, Mirghani H, Péré H, Badoual C. 2020. HPV detection in head and neck squamous cell carcinomas: what is the issue? Front Oncol 10:1751. doi:10.3389/fonc.2020.01751. 33042820 PMC7523032

[21] Atighechi S, Ahmadpour Baghdadabad MR, Mirvakili SA, Sheikhha MH, Baradaranfar MH, Dadgarnia MH, Behniafard N. 2014. Human papilloma virus and nasopharyngeal carcinoma: pathology, prognosis, recurrence and mortality of the disease. Exp Oncol 36:215–216. 25265358

[22] Nikakhlagh S, Saki N, Shoar MH, Sartipipor A, Saki S. 2012. Incidence of etiologic factors in squamous cell carcinoma of head and neck in ahvaz. Iran J Otorhinolaryngol 24:85–90. 24303391 PMC3846216

[23] Sahebjamiee M, Sand L, Karimi S, Biettolahi JM, Jabalameli F, Jalouli J. 2015. Prevalence of human papillomavirus in oral lichen planus in an Iranian cohort. J Oral Maxillofac Pathol 19:170–174. doi:10.4103/0973-029X.164528. 26604492 PMC4611924

[24] Asvadi KI, Seifi SH, Dolatkhah R, Sakhinia E, Dastgiri S, Ebrahimi A, Lotfy A, Esmaeili HAGMMN, Sh H, Haggi AAMN. 2012. Human papilloma virus in head and neck squamous cell cancer. Iran J Cancer Prev 5:21–26. 25780535 PMC4352522

[25] Ursu RG, Danciu M, Spiridon IA, Ridder R, Rehm S, Maffini F, McKay-Chopin S, Carreira C, Lucas E, Costan VV, Popescu E, Cobzeanu B, Ghetu N, Iancu LS, Tommasino M, Pawlita M, Holzinger D, Gheit T. 2018. Role of mucosal high-risk human papillomavirus types in head and neck cancers in Romania. PLoS One 13:e0199663. doi:10.1371/journal.pone.0199663. 29940024 PMC6016945

[26] Bychkov VA, Nikitina EG, Ibragimova MK, Kaigorodova EV, Choinzonov EL, Litviakov NV. 2016. Comprehensive meta-analytical summary on human papillomavirus association with head and neck cancer. Exp Oncol 38:68–72. doi:10.31768/2312-8852.2016.38(2):68-72. 27356572

[27] McKiernan J, Thom B. 2016. Human papillomavirus-related oropharyngeal cancer: a review of nursing considerations. Am J Nurs 116:34–43. doi:10.1097/01.NAJ.0000490170.91898.13. 27428508

[28] Schmitt M, Bravo IG, Snijders PJ, Gissmann L, Pawlita M, Waterboer T. 2006. Bead-based multiplex genotyping of human papillomaviruses. J Clin Microbiol 44:504–512. doi:10.1128/JCM.44.2.504-512.2006. 16455905 PMC1392679

[29] Ozaki S, Kato K, Abe Y, Hara H, Kubota H, Kubushiro K, Kawahara E, Inoue M. 2014. Analytical performance of newly developed multiplex human papillomavirus genotyping assay using Luminex xMAP™ technology (Mebgen™ HPV Kit). J Virol Methods 204:73–80. doi:10.1016/j.jviromet.2014.04.010. 24768623

[30] Nunes EM, Sudenga SL, Gheit T, Tommasino M, Baggio ML, Ferreira S, Galan L, Silva RC, Pierce Campbell CM, Lazcano-Ponce E, Giuliano AR, Villa LL, Sichero L, HIM Study group. 2016. Diversity of beta-papillomavirus at anogenital and oral anatomic sites of men: the HIM study. Virology 495:33–41. doi:10.1016/j.virol.2016.04.031. 27161202 PMC4949595

[31] Razavi Nikoo H, Ardebili A, Ravanshad M, Rezaei F, Teimoori A, Khanizadeh S, Pouriayevali MH, Ajorloo M. 2017. E6-specific detection and typing of human papillomaviruses in oral cavity specimens from Iranian patients. Iran Biomed J 21:411–416. 28460428 10.18869/acadpub.ibj.21.6.411 PMC5572438

[32] de la Cour CD, Sperling CD, Belmonte F, Syrjänen S, Verdoodt F, Kjaer SK. 2020. Prevalence of human papillomavirus in oral epithelial dysplasia: systematic review and meta-analysis. Head Neck 42:2975–2984. doi:10.1002/hed.26330. 32573035

[33] Anonymous. 2007. Human papillomaviruses. IARC Monogr Eval Carcinog Risks Hum 90:1–636. 18354839 PMC4781057

[34] Smelov V, Muwonge R, Sokolova O, McKay-Chopin S, Eklund C, Komyakov B, Gheit T. 2018. Beta and gamma human papillomaviruses in anal and genital sites among men: prevalence and determinants. Sci Rep 8:8241. doi:10.1038/s41598-018-26589-w. 29844517 PMC5974254

[35] Paolini F, Rizzo C, Sperduti I, Pichi B, Mafera B, Rahimi SS, Vigili MG, Venuti A. 2013. Both mucosal and cutaneous papillomaviruses are in the oral cavity but only alpha genus seems to be associated with cancer. J Clin Virol 56:72–76. doi:10.1016/j.jcv.2012.09.016. 23092620

[36] Hadji M, Rashidian H, Marzban M, Gholipour M, Naghibzadeh-Tahami A, Mohebbi E, Ebrahimi E, Hosseini B, Haghdoost AA, Rezaianzadeh A, Rahimi-Movaghar A, Moradi A, Seyyedsalehi MS, Shirkoohi R, Poustchi H, Eghtesad S, Najafi F, Safari-Faramani R, Alizadeh-Navaei R, Ansari Moghadam AR, Bakhshi M, Nejatizadeh A, Mahmudi M, Shahid-Sales S, Ahmadi-Simab S, Nabavian O, Boffetta P, Pukkala E, Weiderpass E, Kamangar F, Zendehdel K. 2021. The Iranian Study of Opium and Cancer (IROPICAN): rationale, design, and initial findings. Arch Iran Med 24:167–176. doi:10.34172/aim.2021.27. 33878874

[37] Muschik D, Braspenning-Wesch I, Stockfleth E, Rösl F, Hofmann TG, Nindl I. 2011. Cutaneous HPV23 E6 prevents p53 phosphorylation through interaction with HIPK2. PLoS One 6:e27655. doi:10.1371/journal.pone.0027655. 22110707 PMC3218003

[38] Viarisio D, Müller-Decker K, Accardi R, Robitaille A, Dürst M, Beer K, Jansen L, Flechtenmacher C, Bozza M, Harbottle R, Voegele C, Ardin M, Zavadil J, Caldeira S, Gissmann L, Tommasino M. 2018. Beta HPV38 oncoproteins act with a hit-and-run mechanism in ultraviolet radiation-induced skin carcinogenesis in mice. PLoS Pathog 14:e1006783. doi:10.1371/journal.ppat.1006783. 29324843 PMC5764406

[39] Bzhalava D, Guan P, Franceschi S, Dillner J, Clifford G. 2013. A systematic review of the prevalence of mucosal and cutaneous human papillomavirus types. Virology 445:224–231. doi:10.1016/j.virol.2013.07.015. 23928291

[40] Meiring TL, Mbulawa ZZA, Lesosky M, Coetzee D, Williamson AL. 2017. High diversity of alpha, beta and gamma human papillomaviruses in genital samples from HIV-negative and HIV-positive heterosexual South African men. Papillomavirus Res 3:160–167. doi:10.1016/j.pvr.2017.05.001. 28720451 PMC5883241

[41] Gheit T, Rollo F, Brancaccio RN, Robitaille A, Galati L, Giuliani M, Latini A, Pichi B, Benevolo M, Cuenin C, McKay-Chopin S, Pellini R, Cristaudo A, Morrone A, Tommasino M, Donà MG. 2020. Oral infection by mucosal and cutaneous human papillomaviruses in the men who have sex with men from the OHMAR study. Viruses 12:899. doi:10.3390/v12080899. 32824507 PMC7472018

[42] Mohebbi E, Hadji M, Rashidian H, Rezaianzadeh A, Marzban M, Haghdoost AA, Naghibzadeh Tahami A, Moradi A, Gholipour M, Najafi F, Safari-Faramani R, Alizadeh-Navaei R, Ansari-Moghaddam A, Bakhshi M, Nejatizadeh A, Mahmoudi M, Shahidsales S, Ahmadi-Simab S, Arabi Mianroodi AA, Seyyedsalehi MS, Hosseini B, Peyghambari V, Shirkhoda M, Shirkoohi R, Ebrahimi E, Manifar S, Mohagheghi MA, Rozek L, Brennan P, Poustchi H, Etemadi A, Pukkala E, Schüz J, Malekzadeh R, Weiderpass E, Rahimi-Movaghar A, Boffetta P, Kamanagar F, Zendehdel K. 2021. Opium use and the risk of head and neck squamous cell carcinoma. Int J Cancer 148:1066–1076. doi:10.1002/ijc.33289. 32895947

[43] Rashidian H, Hadji M, Marzban M, Gholipour M, Rahimi-Movaghar A, Kamangar F, Malekzadeh R, Weiderpass E, Rezaianzadeh A, Moradi A, Babhadi-Ashar N, Ghiasvand R, Khavari-Daneshvar H, Haghdoost AA, Zendehdel K. 2017. Sensitivity of self-reported opioid use in case-control studies: healthy individuals versus hospitalized patients. PLoS One 12:e0183017. doi:10.1371/journal.pone.0183017. 28854228 PMC5576653

[44] Gheit T, Billoud G, de Koning MN, Gemignani F, Forslund O, Sylla BS, Vaccarella S, Franceschi S, Landi S, Quint WG, Canzian F, Tommasino M. 2007. Development of a sensitive and specific multiplex PCR method combined with DNA microarray primer extension to detect Betapapillomavirus types. J Clin Microbiol 45:2537–2544. doi:10.1128/JCM.00747-07. 17581938 PMC1951219

[45] Schmitt M, Dondog B, Waterboer T, Pawlita M, Tommasino M, Gheit T. 2010. Abundance of multiple high-risk human papillomavirus (HPV) infections found in cervical cells analyzed by use of an ultrasensitive HPV genotyping assay. J Clin Microbiol 48:143–149. doi:10.1128/JCM.00991-09. 19864475 PMC2812266

[46] Mokili JL, Dutilh BE, Lim YW, Schneider BS, Taylor T, Haynes MR, Metzgar D, Myers CA, Blair PJ, Nosrat B, Wolfe ND, Rohwer F. 2013. Identification of a novel human papillomavirus by metagenomic analysis of samples from patients with febrile respiratory illness. PLoS One 8:e58404. doi:10.1371/journal.pone.0058404. 23554892 PMC3600855

[47] Jewell NP. 2003. Statistics for epidemiology. Chapman and Hall/CRC.

