# Supplementary material for: Human Papillomavirus and Risk of Head and Neck Squamous Cell Carcinoma in Iran
Source: Microbiol Spectr. 2022 Jun 16;10(4):e00117-22. doi: 10.1128/spectrum.00117-22 (PMC9431561; doi:10.1128/spectrum.00117-22)
Supplement: Supplemental file 1 — Supplemental material. Download spectrum.00117-22-s0001.pdf, PDF file, 0.1 MB [file spectrum.00117-22-s0001.pdf]

Supplementary Table 1: Characteristics of the 13 participants who were positive for α-HPVs among 498 patients and 242 matched controls

No.	Participant	Cancer site (ICD-O code)	Age	Gender	α–HPV type*	β-HPVs, and γ-HPVs status	Tobacco user
1	Control	-	61	Male	HPV58	Negative	Regular user
2	Control	-	51	Male	HPV53	β -HPV-positive	Regular user
3	Control	-	56	Male	HPV18	β -HPV-positive	Regular user
4	Control	-	57	Male	HPV16	β -HPV-positive	Regular user
5	Control	-	38	Male	HPV56	Negative	Regular user
6	Control	-	67	Male	HPV70	Negative	Regular user
7	Control	-	50	Female	HPV16	Negative	Non-user
8	Case	Other tongue (C02.3)	70	Male	HPV58	γ-HPV-positive	Regular user
9	Case	Larynx (C32.0)	70	Male	HPV6	Negative	Regular user
10	Case	HNC, NOS (C76.0)	50	Male	HPV16	β -HPV-positive	Non-user
11	Case	Larynx (C32.0)	45	Male	HPV16	β -HPV-positive<br>γ -HPV-positive	Non-user
12	Case	HNC, NOS (C14.8)	55	Female	HPV11, HPV82	β -HPV-positive	Non-user
13	Case	Other unspecified of the mouth (C06.9)	59	Male	HPV16	β -HPV-positive	Non-user

*The following α-HPV types were not detected in oral rinse samples of patients and controls: HPV26, HPV31, HPV33, HPV35, HPV39, HPV45, HPV51, HPV52, HPV59, HPV66, HPV68, and HPV73. Multiple α-HPV infections were detected in Case 12.
